# Continuous passive motion and physical therapy (CPM) versus physical therapy (PT) versus delayed physical therapy (DPT) after surgical release for elbow contractures; a study protocol for a prospective randomized controlled trial

**DOI:** 10.1186/s12891-017-1854-0

**Published:** 2017-11-22

**Authors:** Jetske Viveen, Job N. Doornberg, Izaak F. Kodde, Pjotr Goossens, Koen L. M. Koenraadt, Bertram The, Denise Eygendaal

**Affiliations:** 1 Department of Orthopaedic Surgery, Amphia Hospital, PO box 90158, Molengracht 21, 4818, CK Breda, The Netherlands; 2 Department of Orthopaedic Surgery, Flinders University, Bedford Park SA, Adelaide, 5042 Australia; 3 Department of Orthopaedic Surgery, University of Amsterdam, Meibergdreef 9, 1105 AZ Amsterdam-Zuidoost, The Netherlands; 4 Foundation for Orthopaedic Research, Care & Education, Amphia Hospital, 4818 , CK Breda, The Netherlands

**Keywords:** Stiff elbow, Operation, Surgery, Rehabilitation, Continuous passive motion, Physical therapy, Randomized controlled trial

## Abstract

**Background:**

The elbow is prone to stiffness after trauma. To regain functional elbow motion several conservative- and surgical treatment options are available. Conservative treatment includes physical therapy, intra-articular injections with corticosteroids and a static progressive or dynamic splinting program. If conservative treatment fails, an operative release of the posttraumatic stiff elbow is often performed. The best Evidence-Based rehabilitation protocol for patients after an operative release is unknown to date and differs per surgeon, hospital and country. Options include early- or delayed motion supervised by a physical therapist, immediate continuous passive motion (CPM), (night) splinting and a static progressive or dynamic splinting program.

**Methods/design:**

The SET-Study (Stiff Elbow Trial) is a single-centre, prospective, randomized controlled trial. The primary objective of this study is to compare the active Range of Motion (ROM) (flexion arc and rotational arc) twelve months after surgery between three groups. The first group will receive in-hospital CPM in combination with early motion Physical Therapy (PT) supervised by a physical therapist, the second group will receive only in-hospital early motion PT supervised by a physical therapist and the third group will receive outpatient supervised PT from postoperative day seven till ten. Secondary outcome measures will be Patient Reported Outcome Measures (PROMs) including the Mayo Elbow Performance Score (MEPS), the Oxford Elbow Score (OES), the quick Disabilities of Arm, Shoulder and Hand (qDASH) score, Visual Analogue pain Scale in rest and activity (VAS), Pain Catastrophizing Scale (PCS), the Short Form (SF)-36, the Centre for Epidemiological Studies Depression Scale Revised (CESD-R) and the Work Rehabilitation Questionnaire (WORQ) for the upper limb.

**Discussion:**

A successful completion of this trial will provide evidence on the best rehabilitation protocol in order to (re)gain optimal motion after surgical release of the stiff elbow.

**Trial registration:**

The trial is registered at the Dutch Trial Register: NTR6067, 31–8-2016.

## Background

All types of post-operative treatment rehabilitation protocols for the elbow are used all over the world: from three in-hospital days of costly continuous passive motion (CPM) and physical therapy (PT) to an outpatient one-day procedure with delayed PT.

The posttraumatic elbow is prone to stiffness [1–6]. After trauma, up to 12 % of all elbows end up with a decreased range of motion (ROM) requiring surgical release [7], however the etiopathogenesis remains largely unknown [6]. According to Morrey and colleagues, most activities during the day can be accomplished with 100 degrees of ROM regarding to flexion-extension arc (30 to 130) and 100 degrees of forearm rotation (50 to 50). [8] Nevertheless, more recently, others reported that functional elbow ROM necessary for activities of daily living may be greater than previously concluded. Contemporary tasks, i.e. as using a computer mouse and keyboard, appear to require greater pronation than drinking from a glass, and using a cellular telephone usually requires greater flexion than for example eating with fork and knife [9].

To regain functional elbow motion several conservative and surgical treatment options are available [6]. Surgical contracture release can be performed open or arthroscopically depending on the type of deformity of the posttraumatic elbow and surgeons’ preference. The results of both techniques are largely comparable, but the amount of complications seems to rise with the extent of the surgical procedure [1, 10, 11]. Recurrence of elbow stiffness up to 37% remains a point of concern and is unrelated to the techniques used to date [10, 12].

Post-operative rehabilitation protocols after operative release for a patient with a posttraumatic stiff elbow include: PT, CPM or a static progressive or dynamic splinting program as prospectively studied by our group [12–14]. In a retrospective study, Lindenhovius et al. previously found that CPM may be redundant [15]. Moreover, from basic science perspective, it theoretically seems that due to early expression of myofibroblasts after trauma that are susceptible to traction (i.e. biomechanical stimuli), it seems that early motion or traction as provided by CPM should be avoided until after the acute phase in order to prohibit the myofibroblasts to produce abundant extra-cellular matrix. This hypothesis is theoretical, based on preliminary histology studies by our group [16]. In contrast to CPM and splinting programs, in all previous described studies physical therapy plays a role and is therefore always included in rehabilitation protocols.

However, the best Evidence-based rehabilitation protocol regarding additional treatment and timing of physical therapy for patients after an operative release is unknown and differs per surgeon, hospital and country. The most recent review on this topic does not show a clear clinical benefit of any of the respective post-operative rehabilitation protocols [6]. However, no randomized controlled trials were included. Therefore, we study which type of rehabilitation protocol after elbow capsule release at one year after surgery is most effective in terms of range of motion (ROM) in a randomized controlled trial. The potential health care efficiency gain consists of more homogeneity in rehabilitation after elbow capsule release and less costs regarding in-hospital stay. Hence, unnecessary treatments could be avoided and a more universal treatment can be established.

## Methods

### Study design

The SET-study is a three-arm, prospective, single-centre, randomized controlled trial with twelve months of follow-up. One teaching hospital in the Netherlands will participate. Patient will be included in one of the three post-surgical release rehabilitation arms after index surgery for stiff elbow:

1. In-hospital Continuous Passive Motion with Early Motion Supervised Physical Therapy (CPM)
2. In-hospital Early Motion Supervised Physical Therapy (PT)
3. Outpatient Supervised Physical Therapy from postoperative day 7–10 (DPT)

In our hospital, the gold standard as rehabilitation protocol after capsule release of the stiff elbow includes CPM with physical therapy. Therefore, the control-group of our study is group one (CPM-group).

### Recruitment and consent

All patients presenting to our outpatient Orthopaedic Elbow clinic with a posttraumatic stiff elbow that plateaus in their post-fracture rehabilitation program after more than 6 months after trauma, who meet the inclusion criteria will be invited to participate in the trial. Each patient will receive a Computed Tomography (CT-) scan, since this is part of our usual care.

The treating surgeon or resident will introduce the trial to the patient and address the patient’s questions. Information will be handed to the patient to read at home. If the patient is willing to participate, written informed consent is required and will be obtained. Participants will be given a copy of the consent form and will be informed that their participation is voluntary and that they can withdraw from the study at any time. Participants may take as long as they like to consider participation, provided that they still meet all eligibility criteria stated below.

After providing informed consent, each patient will be randomly assigned to the CPM- PT- or DPT-group on the basis of a random sequence determined by a computerized random-number generator based on our enrolment goal of 90 patients (Castor Electronic Data Capture (EDC), Ciwit BV, Amsterdam, the Netherlands).

### Study population

Patients with the following inclusion criteria are eligible for enrolment*:*

- Age between 18 and 65 years
- Flexion-extension arc of <100° or a contracture >30° compared to contralateral side
- Open or arthroscopic surgical treatment received
- More than 6 months after trauma
- Unsuccessful conservative treatment
- Able to read and write in Dutch
- Provision of informed consent by patient

If any of the following criteria apply, patients will be excluded:

- Inflammatory diseases (i.e. rheumatoid arthritis, psoriatic arthritis, or reactive arthritis)
- Patients with any other elbow pathology (i.e. spastic contracture)
- Neck pain or shoulder pain or other chronic widespread pain syndromes
- Wound problems
- Inability to cooperate with a structures rehabilitation protocol
- Burn-related contractures
- A total elbow or interposition arthroplasty (either planned or in place)

### Intervention

Patients that are assigned to the ‘CPM’ group will receive in-hospital CPM during one hour in combination with supervised physical therapy, both three times a day, the first three days (in hospital), starting two till four hours after surgery depending on anaesthesia and supervised physical therapy from day three till day 14 postoperative (at home). Patients that are assigned to the ‘PT’ group will receive in-hospital supervised physical therapy three times a day the first three days, start two till four hours after surgery and supervised physical therapy from day three till day 14 postoperative at home. Patients that are assigned to the ‘DPT’ group will receive outpatient physical therapy from postoperative day 7–10 till 14 and will be discharged from the hospital immediately after surgery. In-hospital physical therapy includes both active and passive exercises, manual manipulation of rotations and angular mobilization of the ulnohumeral joint, with a duration of 10 min three times a day; moreover, patients will be invited to practice themselves as well. Thereby, the patient will be provided wrist- and hand exercises to improve circulation and to prevent oedema.

After discharge from the hospital, all patients of the three groups are handed out the same physical therapy program for the first six months. After the first 14 days, all patients will continue with physical therapy, which is recommended three till four times a week the first 4 months. Thereafter, the amount of physical therapy sessions can be reduced, but sessions themselves can be extended with use of weights. Patients are allowed to choose an outpatient physical therapist by themselves. In addition, patients are asked to fill in Patient Reported Outcome Measures (PROMs) during every appointment at the outpatient clinic.

### Outcome measures

The primary outcome measure is the active ROM (flexion, extension, pronation, supination) of both elbow joints, measured with a universal goniometer, one year after surgery. The references for measuring the ROM are the acromion and the styloid process of the radius, with the lateral epicondyle as central point of the rotation.

Secondary outcome measures are PROMs which consist of;

- The Oxford Elbow Score (OES) [17, 18], which reflects both function and pain following elbow surgery. The OES consists of three domains; pain, function and social-psychological. Each domain comprises of 4 questions with 5 response options per question. Each response is scored 0 to 4, with 0 representing greater severity. Scores for each domain are calculated as the sum of each individual item score within that domain. These scores are then converted to a metric score between 0 and 100 (a lower score represents greater severity). The Minimal Clinically Important Difference (MCID) for the OES is 10 points [19].
- The Mayo Elbow Performance Score (MEPS) [20], which is based on 4 domains (pain, range of motion, stability and elbow function). A total score between 90 and the maximum 100 points is considered excellent; 75–89 is good; 60–75 is fair and less than 60 points is poor. The MCID for the MEPS is 15 points [21].
- The quick Disabilities of Arm, Shoulder and Hand (qDASH) score [22–24], which is scored in two components: the disability/symptom section (11 items, scored 1–5) and the optional high-performance sport/music or work modules (four items, scored 1–5). The quick-DASH is designed to measure physical function and symptoms in patients with any or several musculoskeletal disorders of the upper limb. The MCID for the qDASH is 10 points [25].
- The Visual Analogue pain Scale in rest and activity (VAS) [26], which is a one-dimensional measure of pain intensity scored between 0 and 100. 0 is no pain and 100 is the maximum of pain. The MCID for the VAS 0–100 is 14 points [27].
- The Pain Catastrophizing Scale (PCS) [28], which consists of 13 items reflecting catastrophic thinking in relation to pain. The PCS total scores ranges from 0 to 52, in which a higher score is related to an experienced higher level of physical and emotional distress associated with the pain condition. The MCID for the PSC is not established.
- The Short Form (SF)-36 [29], which measures the quality of life. The SF-36 consists of eight scaled scores, which are the weighted sums of the questions in their section. Each scale is directly transformed into a 0–100 scale on the assumption that each question carries equal weight. The lower the score the more disability. The MCID for the SF-36 is not established.
- Centre for Epidemiological Studies Depression Scale Revised (CESD-R) [30], which consists of 20 items, is a screening test for depression and depressive disorder. Patients are asked to fill in how many times they were experiencing specific symptoms (not at all or less than one day = 0, 1–2 days = 1, 3–4 days = 2, 5–7 days = 3, nearly every day for 2 weeks). The sum of the answers in combination with DSM criteria is representative for the severity of the depression. The MCID for the CESD-R is not established.
- The Work Rehabilitation Questionnaire (WORQ) for the upper limb is a questionnaire specific for work-related limitations in patients with upper extremity musculoskeletal disorders. The content includes four categories: exertion, dexterity, handling tools & equipment, and mobility. Patients are asked to fill in how difficult it was to perform several activities while performing their job (caused by symptoms of the affected upper extremity). Patients have to rate their difficulty on a 6-point scale: 0 = not applicable (in my job), 1 = not at all, 2 = slightly, 3 = moderately, 4 = very, 5 = extremely, or I cannot do this. The MCID for the WORQ is not established.

### Study procedures

**Fig. 1:**
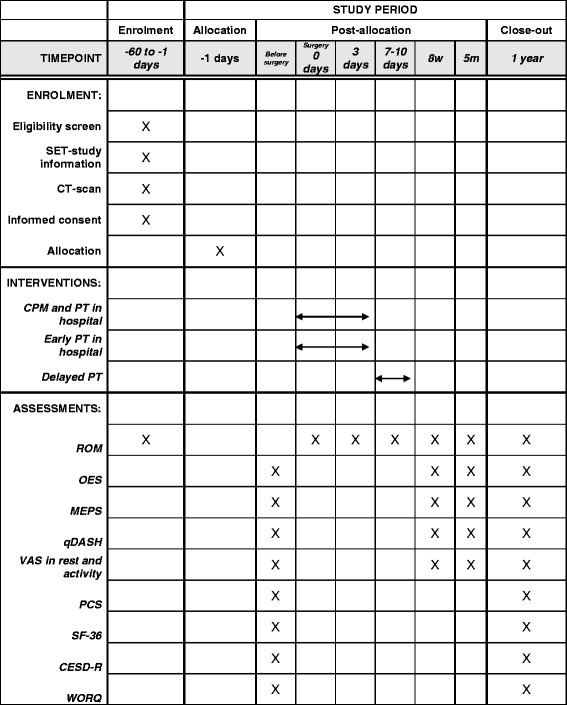
Timeplan study procedures of the SET-study

Clinical assessment will be performed preoperatively (baseline), during surgery, at day three, at day ten, eight weeks, five months and one year after surgery. A link to all (online) questionnaires will be sent to the patients at baseline and one year after surgery. In addition, a link of the qDASH, OES, VAS and MEPS will be sent eight weeks and five months after surgery. An independent and blinded researcher will perform postoperative assessment (Fig. 1).

### Sample size calculation

The primary study question addresses improvement in active elbow flexion-extension arc and pronation-supination arc at one year. A power analysis indicated that a total sample size of 72 patients (24 patients in each cohort) would provide 80% power (b = 0.20, a = 0.05) to detect a clinically relevant difference of ten degrees in improved flexion arc. To account for a possible loss to follow-up of 20 to 25%, we anticipate enrolling 30 patients in each cohort; 90 patients in total for this study.

### Statistical analysis

The data will be presented in line with the SPIRIT statement. Analyses will be performed according to the intention-to-treat principle, according the following example: when a patient crossed over from the ‘CPM’ group, to the ‘PT’ group (i.e. still having supervised physical therapy, but the patient refuses CPM, or does not tolerate CPM), analysis will be according to initial randomization, with this patient in this particular example still being analyzed in the ‘CPM group’ although the patient did not receive CPM, he or she will be analyzed according the intention to treat in this cohort. After completing the trial, a post-hoc power analysis will be performed again, to address and account for alpha post-RCT with known standard deviation in our three study groups.

Patients’ baseline characteristics will be compared between the three groups. Depending on the distribution of the gathered data, Chi-square tests or Fisher’s Exact tests will be used for categorical variables, and ANOVA or Kruskal Wallis tests for continuous variables.

For our primary outcome, the improvement in active elbow flexion-extension arc in degrees after one year, an ANOVA will be performed to compare the three groups. For the other time-points and the other continuous outcome parameters (flexion, extension, flexion contracture, forearm rotation, pronation, supination, qDASH scores, OES scores, MEPS scores, SF-36 scores, CESD-R scores, VAS pain scores, PCS scores and WORQ scores and number of additional surgeries) also the changes compared to the pre-randomization scores will be compared between cohorts using ANOVAs. An alpha correction for multiple comparisons on the same secondary outcome measure will be applied.

Differences in categorical outcome parameters (MEPS) will be compared between the groups at each time-point using the Chi-square test for trend. Furthermore, the association between the arc of elbow flexion and extension and the qDASH scores will be analyzed at enrolment and at the six- and twelve-month evaluations using Spearman correlation. Also in these cases an alpha correction for multiple comparisons on the same secondary outcome measure will be applied.

### Ethical considerations

Based upon current scientific literature, there is no clear preference for one of the treatments regarding the increase of ROM after surgery. The different treatment options are regularly applied for posttraumatic elbow contractures in the participating institution and all surgeons participating in this study are familiar with the procedure. Patients will participate in one of the therapy options. Patients who miss their standard follow-up evaluation will be invited to return for follow-up at no cost to them. The risks and discomfort of participating in this study do not exceed those of standard treatment for this condition. The questionnaires will take 20 min to complete.

Patients in all cohorts are likely to benefit from treatment. The motivation for the study is a potential benefit to all patients with posttraumatic elbow contractures, as we increase our knowledge on optimal treatment of this condition.

### Monitoring and quality assurance

The Institutional Review Board of the principal investigators’ hospital has approved the current study under the number: NL58264.018.16. The information collected during this study will be placed in a research folder and not added to the patient’s medical record unless expressly requested by the patient. Patient data are not directly transferable to the patient as for each patient the hospital patient number is used. All research folders will be filed in Castor EDC, independent of clinical charts or any other medical record in electronic format. Any magnetic or electronic information will be saved in password-protected computers to which only study staff will have access. Only the executive researchers have access to the data.

A member of the study staff will be responsible for monitoring outcomes. No independent monitoring will occur. All investigators and study staff will be responsible for reporting adverse effects to the coordinating investigator. Our coordinating investigator will report adverse events to the ethical committee in accordance with the ethical committee adverse event reporting procedures. The coordinating investigator and the principal investigator are responsible for adherence to all ethical committee rules and guidelines and for the accuracy and completeness of all forms, entries, and informed consent. This algorithm is as described by our group, which we use in our hospital [31].

The results of this trial will be described in an article, which will be submitted for publication in an international peer reviewed journal by the coordinating investigator.

## Discussion

To date there have been no prospective randomized trials comparing rehabilitation protocols after surgical release of a stiff elbow. The SET-study will compare management of these rehabilitation protocols by using in-hospital CPM in combination with early motion supervised PT, in-hospital early motion supervised PT without CPM and outpatient delayed supervised PT. Based upon current scientific literature, there is no clear preference for one of the treatments regarding the increase of ROM after surgery. CPM in elbow surgery may be redundant [15] and also in other joints such as the knee, the use of CPM as after treatment seems ineffective and unnecessary [32].

The different treatment options are regularly applied for posttraumatic elbow contractures in the participating institution and all surgeons participating in this study are familiar with the procedure. Patients in all cohorts are likely to benefit from treatment. The motivation for the study is that unnecessary treatment burden for patients (prolonged hospital stay, because of CPM sessions) as well as redundant costs for society can be avoided, a more universal Evidence-Based method of treatment can be established and the quality of the care can be improved. Regarding to potentially less healthcare costs, a cost-effectiveness analysis will be performed in a separate study.

Patient enrolment will start in March 2017 and we expect to enroll 4 patients per month. Considering the one-year follow-up, publication of data will be expected in 2020.

## Abbreviations

CESD-R: Centre for Epidemiologic Studies Depression Scale Revised
CPM: Continuous Passive Motion
CT: Computed Tomography
DASH: Disabilities of the arm, shoulder and hand score
DPT: Delayed Physical Therapy
DSM: Diagnostic and Statistical Manual of Mental Disorders
EDC: Electronic Data Capture
MCID: Minimal Clinically Important Difference
MEPS: Mayo Elbow Performance Score
NTR: Netherlands Trial Registry (in Dutch: Nederlands Trial Register)
OES: Oxford Elbow Score
PCS: Pain Catastrophizing Scale
PROMs: Patient Reported Outcome Measures
PT: Physical Therapy
ROM: Range of motion
SET: Stiff Elbow Trial
SF: Short Form
SPSS: Statistical Package for the Social Sciences
VAS: Visual Analogue Score
WORQ: Work Related Questionnaire
